# The Influence of Polymer Fibers on the Properties of Foam Concrete with a Complex Nanomodifying Additive: Finite Element Analysis and Experimental Study

**DOI:** 10.3390/polym18080988

**Published:** 2026-04-18

**Authors:** Alexey N. Beskopylny, Sergey A. Stel’makh, Evgenii M. Shcherban’, Diana M. Shakhalieva, Andrei Chernil’nik, Ivan Panfilov, Nikita Beskopylny, Zhipeng Li, Weiyi Kong

**Affiliations:** 1 Department of Transport Systems, Faculty of Roads and Transport Systems, Don State Technical University, 344003 Rostov-on-Don, Russia; nbeskopylnyi@donstu.ru; 2 Department of Unique Buildings and Construction Engineering, Don State Technical University, 344003 Rostov-on-Don, Russia; sstelmah@donstu.ru (S.A.S.); achernilnik@donstu.ru (A.C.); 3 Department of Engineering Geometry and Computer Graphics, Don State Technical University, 344003 Rostov-on-Don, Russia; etsherban@donstu.ru; 4 Department of Design, Don State Technical University, 344003 Rostov-on-Don, Russia; delshaeva@donstu.ru; 5 Department of Theoretical and Applied Mechanics, Agribusiness Faculty, Don State Technical University, Gagarin Square, 344003 Rostov-on-Don, Russia; panfilov.i@gs.donstu.ru; 6 School of Transportation and Civil Engineering, Shandong Jiaotong University, Jinan 250357, China; lizhipeng@sdjtu.edu.cn (Z.L.); kongweiyi@sdjtu.edu.cn (W.K.)

**Keywords:** polymer fiber, foam concrete, fiber foam concrete, polypropylene fiber, carbon fiber, compression strength, flexural strength, interface bond strength

## Abstract

Modern construction extensively utilizes foam concrete (FC) because of its distinct characteristics. However, its application is limited by its low strength properties. Developing high-strength FC by strengthening the matrix with various additives and incorporating various types of fibers into the composition is one of the most rational trends, consistent with the concept of sustainable and environmentally friendly construction. This study explores the impact of diverse polymer fibers on the strength and deformation characteristics of fiber-reinforced foam concrete (FRFC). The concrete’s matrix is strengthened by a composite nanomodifying additive. A FEM model was developed, and experimental studies of the compressive and flexural strength of FRFC were conducted. In the numerical study, the FC matrix is described by the Menetrey-Willam model. Parameter calibration and model verification demonstrated good agreement with experimental data. Experiments and numerical simulations proved that polypropylene fibers enhance compressive strength by as much as 20% and flexural strength by 80%. The stress–strain condition of FRFC was numerically analyzed, considering the influence of steel, carbon, and glass fibers. It was shown that high-modulus polymer fibers quickly lose their adhesive properties and impair the deformation properties of the composite compared to polypropylene fibers.

## 1. Introduction

Recently, a growing demand for lightweight and energy-efficient building materials has occurred, one of which is FC. The global FC (cellular concrete) market is demonstrating steady growth, driven by the construction industry’s shift toward environmentally friendly and energy-efficient materials [1,2]. FC fits well with the environmental agenda and the trend toward energy efficiency. It is actively being introduced as a material with high thermal insulation properties, which helps reduce the heating and cooling costs of buildings and meet green standards [3]. The growing demand for lightweight materials for high-rise buildings and infrastructure facilities is driving the use of FC to reduce foundation loads. From a technological perspective, the development of high-strength FC [4] and the introduction of automated production technologies (including additive manufacturing and modular construction) are expanding its range of applications [5].

The material is increasingly used to fill voids and insulate roofs and floors as a more cost-effective and durable alternative to traditional insulation [6]. Foam concrete blocks are used to construct load-bearing walls (usually up to 2–3 stories) and internal structures. The blocks are large, making construction several times faster than brick construction. Foam concrete is used in monolithic construction: for this, liquid foam concrete is poured into formwork directly on the construction site. This allows for the creation of seamless structures with excellent water resistance.

The relevance of research aimed at improving the properties of foam concrete is confirmed by its practical significance. In 2024, the global lightweight concrete market was estimated at $739–743 million. It is projected to reach $1.08 billion by 2030, growing at a rate of approximately 6.3–6.5%. Accordingly, improving the performance properties of lightweight concrete by adding various types of fibers is a pressing issue of both practical and scientific interest.

The unique properties of FC are in conflict with one another: the higher the thermal conductivity, the lower the strength. In practice, it is important to balance the key properties of FC, particularly compressive strength and thermal conductivity. This is achieved by strengthening the cement matrix with various additives, nanomodifiers, and dispersion reinforcement [7].

The impact of nanosilica on FC properties, with the inclusion of polystyrene, was studied by the authors [8,9,10]. The combination of nanosilica and polystyrene was demonstrated to offer excellent thermal insulation, enhance water permeability, and guarantee the robustness of the cement matrix. The mechanical strength and durability of lightweight concrete were enhanced by the addition of 3.5% silica, while its performance remained unaffected. In [11], the authors conducted a sensitivity analysis of a machine learning model for predicting the thermal conductivity of FC using an extreme gradient boosting algorithm. Covariance matrix analysis showed that air content has the largest contribution to thermal conductivity properties (54%), while nanosilica content has an effect of 11%. However, it remains unclear to what extent FC strength decreases with increasing pore number and size in foam concrete. In [12], a technology for producing FC based on alkali-activated copper slag was studied. The concentration of the alkali prepared from sodium hydroxide granules and sodium silicate solution was 6%, the proportion of silica varied from 0.5 to 1.5%. By incorporating 1.5% microsilica, the compressive strength reached its peak, ranging from approximately 3 MPa to 4.75 MPa. The study in [13] focused on the partial replacement of cement using bentonite. According to the authors, bentonite FC features a fine-pored structure. FC with 0.2% bentonite showed a 20% rise in compressive strength, a 25% better frost resistance in sulfate conditions, and a 33.3% greater resistance to drying–wetting cycles. The primary reason for bentonite’s effectiveness in strengthening FC lies in its silica’s layered tetrahedral structure, which exhibits a powerful affinity for water. Overall, numerous studies [14,15,16] demonstrate the positive impact of the pozzolanic reaction and the formation of additional calcium silicate hydroxides (C-S-H) on the structure and properties of FC, making the cement stone denser and stronger. Due to the formation of a dense C-S-H gel, the pore walls become stiffer and less prone to microcracks. It makes it possible to produce strong FC at low densities. Another important approach to improving the properties of FC is dispersion reinforcement with polymer materials [17,18,19,20]. Polymer fibers, as a reinforcing component of FC, significantly improve its properties.

Let us consider the most common types of polymer fibers and their effect on structure.

- Polypropylene fibers [21,22,23] are a prevalent and economical choice for enhancing the structure and characteristics of FC. Crack resistance is enhanced, shrinkage is reduced, and frost resistance is increased by polypropylene fibers. Their mechanism of action is based on the creation of a spatial network that prevents the development of microcracks.
- Polyethylene fibers [24,25] have high chemical resistance and durability. Polyethylene fibers contribute to increased impact strength and resistance to aggressive environments.
- Synthetic fibers (basalt, carbon) [26,27] are used to produce durable FC. These fibers provide a significant increase in compressive and flexural strength, as well as increased fire resistance.

Optimal reinforcement parameters depend on the type, geometry, and properties of the fiber. Typically, fiber content ranges from 0.1 to 1.0% by volume [28,29], significantly improving properties. Conversely, a high fiber amount may result in lower workability and strength.

Combining various nanoparticles and functional additives can produce a synergistic effect, where the overall improvement in properties exceeds the sum of the improvements from each component individually.

Discussions regarding the creation of novel wall materials that are low in carbon, light, and offer good thermal insulation can be found in [30]. The study examined the effect of fiberglass on the properties of geopolymer foamed concrete produced by chemical foaming. The results showed that with a fiber length of 9 mm and a fiber content of 0.3%, the geopolymer foamed concrete had “a density of 710 kg/m^3^, a compressive strength of approximately 5 MPa (>20% increase), and a thermal conductivity of 0.07–0.08 W/(m K)” (approximately a 20% decrease). However, there are several challenges in creating lightweight and durable FC. As an illustration, papers [31,32] found that the flexural strength decreased by 40.0% when the polystyrene content was raised, in contrast to the control. A slight increase in flexural strength to 1.99 MPa was observed due to microsilica. A notable increase in flexural strength was observed because of the inclusion of 1% polypropylene fibers in the mixture. The increase in strength amounted to 33.5%, which is explained by including polypropylene fibers. However, it remains unclear which fibers contribute the greatest increase in strength without sacrificing other properties.

Hybrid reinforcement of FC with steel and polypropylene fibers was employed in [33,34]. Compared to standard FC, these prisms demonstrated greater stress resistance and better load-deflection performance. Steel fibers helped manage crack expansion and enhanced load distribution; meanwhile, polypropylene fibers boosted resistance to micro-cracks. Consequently, the compressive strength of FC with 1% steel fiber and 0.3% polypropylene fiber was 7.33 N/mm^2^ after 28 days, an improvement of 62.16% over conventional FC.

In [35,36,37,38], the authors demonstrate the advantages of FC with different fiber types. Different types of fibers in concrete production provide superior resistance to impact, abrasion, and fracture. It is recognized that extended steel or synthetic fibers can entirely substitute for reinforcement or steel in specific scenarios [39,40,41]. Natural fibers impart various unique properties to FC [42,43,44]. The nature of FC’s properties changes with changes in the geometry, concentration, orientation, and mechanical properties of the fibrous materials.

These studies demonstrate that the selection of optimal dimensions and properties of artificial or natural fibers, as well as their influence on the overall properties of FC, remain unclear. To understand the mechanics of interaction between fibers and the porous structure of FC, numerical analysis of models is used, taking into account loading characteristics and the functional orientation of the structure [31,45,46,47,48]. In [49], a two-dimensional FE model of a sandwich panel filled with FC was developed, taking into account the brittleness of FC and the bond effect between polymer reinforcement and mortar. The model allowed for the study of the nature of interfacial sliding between the concrete and the panel. The study’s authors observed a strong correlation between the findings, proving the FE model’s ability to precisely replicate the failure mechanism and the load–displacement behavior. Although the numerical analysis results appear to closely match the experimental data, the study does not demonstrate how the geometry and mechanical properties of the polymer fibers affect the performance of fiber-reinforced foam concrete. Furthermore, the adhesion of the fibers to the matrix remains unexplored.

In [50], the mechanical behavior of FC was modeled under uniaxial or biaxial compression using cubic specimens. The model development focused on understanding the stress evolution and damage onset in relation to the observed physical events. However, the paper does not show how the characteristics of the polystyrene beads affect the physical and mechanical behavior of EPS concrete. Furthermore, as shown in [31,51,52], the calibration of the Menetrey-Willam or microplane models requires time-consuming experimental studies and performs poorly for three-phase composites such as FRFC. In [31], it was shown that in the softening zone, the model deviates significantly from the experimental data.

The aforementioned gaps led to the formulation of the aim of this work: to develop a numerical finite element model of FRFC with increased strength due to matrix reinforcement with microsilica and alumina nanoparticles, and to conduct a parametric study of the influence of fiber shape and properties on the strength characteristics of FRFC.

Goals of the research:

1. Experimental study of the correlation between the composition, structure, and properties of FRFC with microsilica and alumina nanoparticles;
2. Development of a finite element model of FRFC and calibration of the parameters of the nonlinear behavior of concrete under compression and bending.
3. Identification of patterns in the stress–strain state of FRFC under compressive and bending loads.
4. Examining experimental and numerical data to identify optimal formulation and technological factors affecting FRFC strength.

## 2. Materials and Methods

### 2.1. Materials and Research Sequence

**Figure 1:**
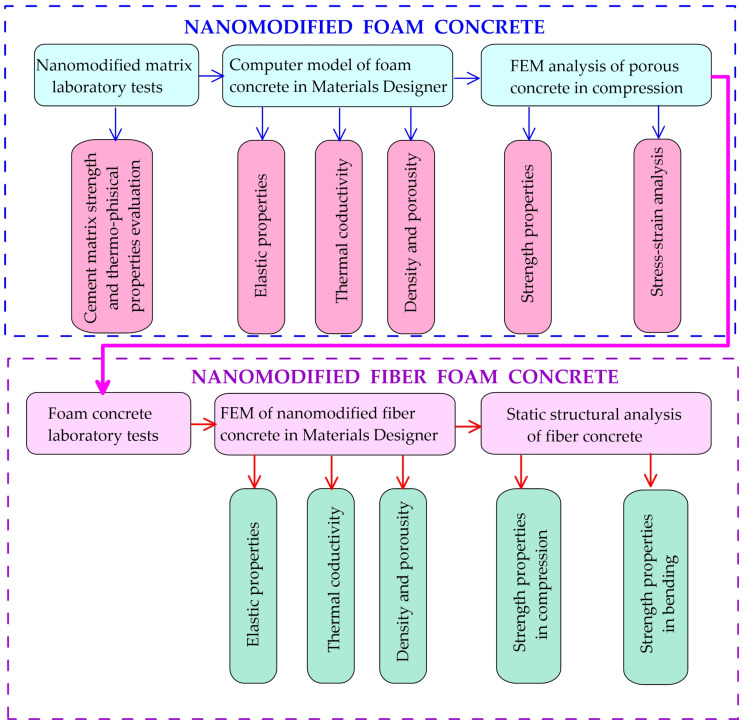
Scheme of sequential experimental and numerical studies.

This article utilizes the methodology of [31], which involves conducting the study in several stages. Figure 1 contains a diagram illustrating the current study.

Experimental FC and FRFC samples were produced using the raw materials detailed in Table 1.

A complex nanomodifying additive (CNA) is a mixture of dry mineral components. To obtain CNA, the mineral components, nanoparticles, and plasticizing additive were mixed in an Activator-4M planetary ball mill (Chemical Engineering Plant LLC, Novosibirsk, Russia) for 2 min at 600 rpm in the following ratio: FA—80%; MS—18%; NA—2%. The plasticizing powder additive C-3 was added at a rate of 0.2% of the final mass of the FA, MS, and NA mixture. Mechanical mixing ensures uniform mixing of all components, increases their specific surface area, and enhances reactivity.

According to the formula in Table 2, experimental FC and FRFC samples were produced by following these primary process steps:

Step 1. Dosing and preparation of raw materials in the required quantities.

Step 2. Load the raw materials into the mixer in the following sequence: PC, QS, water, CNA, and PF.

Step 3. Intensively mix the raw materials until the FC mixture reaches a uniform consistency and is poured into 40 × 40 × 160 mm metal prism molds.

Step 4. Strip the experimental FC and FRFC samples 24 h after production, followed by 27 days of curing before testing. The temperature and relative humidity during sample curing were maintained at 20 ± 2 °C and 60 ± 10%, respectively.

### 2.2. Experimental Research Methods

**Figure 2:**
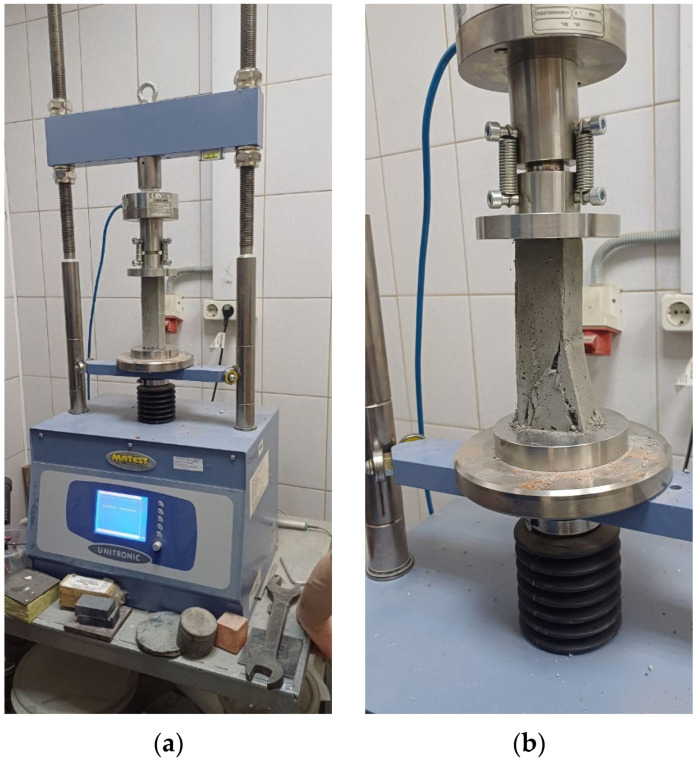
Tests of prismatic strength of FC and FRFC: (**a**) general view of the setup; (**b**) sample after destruction.

**Figure 3:**
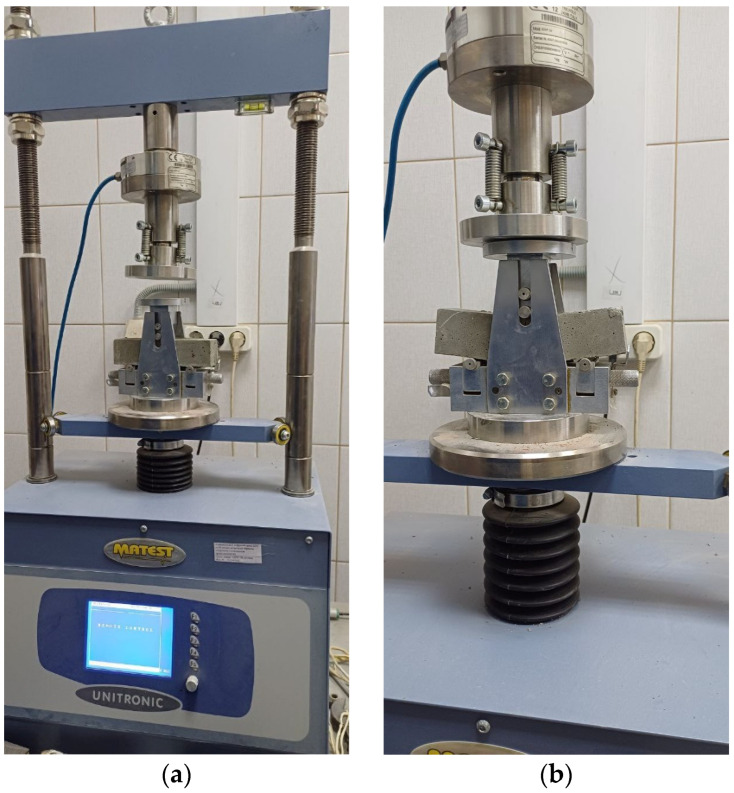
Flexural testing of FC and FRFC: (**a**) general view of the setup; (**b**) sample after testing.

Experimental studies of prismatic strength and flexural strength were conducted in accordance with regulatory documents [53,54,55]. To determine axial compressive strength, experimental FC and FRFC specimens were mounted on the support plates of an S205 testing machine (Matest, Treviolo, Italy), and a load was applied. Main technical characteristics of Matest S205N: ultimate compression/bending load—50 (kN); pressure plate travel speed—(0.01–51) mm/min; loading rate—1–15,000 (N/s). For compression, the loading rate of the samples was 2400 ± 200 N/s. For bending, the loading rate of the samples was 50 ± 10 N/s. The strength of the samples was calculated with an accuracy of 0.1 MPa. To determine flexural strength, experimental FC and FRFC specimens were first mounted on the support elements of the apparatus PII-40 (Promtekhnologii, Saint Petersburg, Russia). The setup with the specimen was positioned on the testing machine’s supports, and then a load was introduced. Photographs of the tests are shown in Figure 2 and Figure 3.

**Figure 4:**
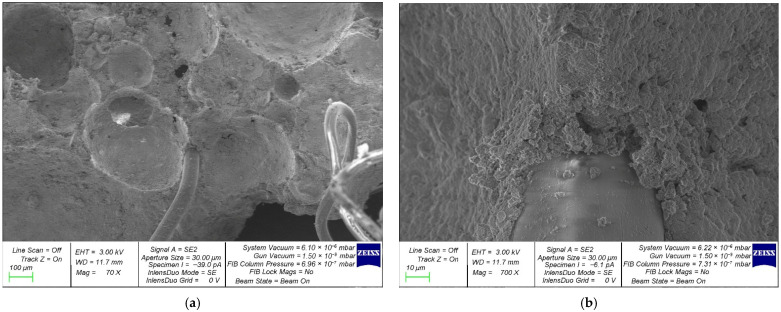
SEM of the FRFC structure at magnifications: (**a**) 70×; (**b**) 700×.

Figure 4 displays FRFC microstructure images acquired via scanning electron microscopy using a ZEISS CrossBeam 340 (Carl Zeiss Microscopy GmbH, Jena, Germany).

The FRFC matrix shown in Figure 4 has a well-organized, homogeneous structure. The internal walls of the pore cells are dense and have a uniform surface. The pore structure of fiber-reinforced foam concrete is represented by closed round pores, interpore partitions, and PFs, which are located at the junctions of the interpore partitions. The zone at the cement matrix–fiber interface is dense and free of significant visible defects. The tight adherence of the fiber to the cement matrix indicates a high level of fiber–matrix adhesion.

### 2.3. FEM Modeling

Materials Designer within ANSYS (version 2022 R1) was used for FRFC modeling. A representative FRFC sample is produced by this module, which then ascertains its functional properties like elastic modulus per axis, thermal conductivity, and density. A fiber diameter of 34 µm was specified in the module, and the inter-fiber angle was selected between 0 and 40° to ensure stochastic fiber order.

For finite element modeling of concrete, a high-order 3D solid element with 20 SOLID186 nodes is used: 14,864 nodes and 3220 elements with a minimum mesh size of 0.1 mm. Mesh dimensionality verification was performed: for the final iteration, the error was less than 0.01%, and further refinement did not change the result. For modeling the reinforcing fibers, the “Reinforcement” model is used: a REINF264 beam element. Each fiber is modeled separately as a rod with only uniaxial stiffness. In the first step, the full 3D geometry with volumetric fibers is constructed in the Ansys (version 2022 R1) MaterialDesigner module, then the volumetric fibers are replaced with the “Reinforcement” model in the geometry editor. For the numerical solution of the nonlinear deformation problem, the “Newton-Raphson” method in the “Unsymmetric” formulation is used. The initial step of the solution is 100, the minimum step is 100, the maximum number of steps is 5000.

**Figure 5:**
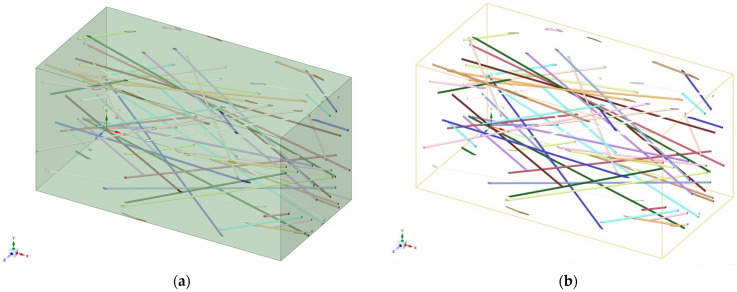
Model of FRFC: (**a**) geometric model; (**b**) fiber arrangement.

Figure 5 shows the geometric models of FRFC and the spatial arrangement of the fibers used in the modeling. Since the Material Design module only produces effective moduli matrices in a linear formulation, this module was used specifically to generate the geometric model of the two-component composite. Finite element modeling was performed in Ansys (version 2022 R1) Mechanical.

Thus, the dimensions of the representative sample were 1.8411 mm × 0.92054 mm × 0.92054 mm, with a polymer fiber content of 3.9 kg/m^3^.

### 2.4. Calibration of the Menetrey-Willam Model

The Menetrey-Willam model is a nonlinear model of concrete deformation and currently provides the most accurate description of the stress–strain state compared to other models, such as the Drucker–Prager model. The model includes elastic constants and parameters characterizing the hardening and softening stages of concrete under various types of loading. Model calibration, which involves selecting parameter values, is a separate task that affects the accuracy of subsequent calculations.

During hardening in the Menetrey-Willam model, the yield surface follows a complex nonlinear relationship denoted as *σ* = *f*(*ε*), and after the plastic potential function reaches a critical value, it exhibits exponential softening. The increment in total strain is the sum of the elastic and plastic components.

(1) $$d\varepsilon =d\varepsilon^{el}+d\varepsilon^{pl}$$

The stresses in the elastic zone are related to the deformation based on the generalized Hooke’s law

(2) $$d\varepsilon^{el}={\left[D\right]}^{-1}d\sigma$$

(3) $${\left[D\right]}^{-1}=\frac{1}{E}\left[\begin{array}{cccccc} 1 & -\nu & -\nu & 0 & 0 & 0\\ -\nu & 1 & -\nu & 0 & 0 & 0\\ -\nu & -\nu & 2\left(1+\nu \right) & 0 & 0 & 0\\ 0 & 0 & 0 & 2\left(1+\nu \right) & 0 & 0\\ 0 & 0 & 0 & 0 & 2\left(1+\nu \right) & 0\\ 0 & 0 & 0 & 0 & 0 & 2\left(1+\nu \right) \end{array}\right]$$

Here *E* is Young’s modulus; *μ* is Poisson’s ratio.

Plastic deformation increases according to the non-associated flow rule, where this increase is linked to the plastic potential Ω.

(4) $$d\varepsilon^{pl}=d\zeta \frac{\partial \Omega }{\partial \sigma }$$

where σ is the stress tensor, ζ is a non-negative plastic multiplier, and Ω is a plastic potential function.

The Menetrey-Willam model takes into account the different properties of concrete under tension and compression, which determines the relationships [31,51,52].

(5) $$\Omega_{c}=\Omega_{ci}+\left(1-\Omega_{ci}\right)\;\sqrt{2\frac{\varepsilon_{c}}{\varepsilon_{c1}^{pl}}-\frac{\varepsilon_{c}^{2}}{\varepsilon_{c1}^{pl\;2}}}$$

where $\Omega_{c}$ is the compression hardening/softening function of concrete, $\Omega_{ci}$ is the stress corresponding to the transition from elastic to plastic deformations, $\varepsilon_{c}$ is the strain, $\varepsilon_{c1}^{pl}$ is the plastic strain corresponding to the peak stress, $\Omega_{c}=\sigma_{c}/f_{c}$, $f_{c}$ where is the compressive strength under uniaxial compression. The branch corresponding to the material softening is divided into two fragments:

(6) $$\left\{\begin{array}{l} \Omega_{c}=1-\left(1-\Omega_{cu}\right)\;{\left(\frac{\varepsilon_{c}-\varepsilon_{c1}^{pl}}{\varepsilon_{c,\lim}^{pl}-\varepsilon_{c1}^{pl}}\right)}^{2},\;\varepsilon_{c1}^{pl}<\varepsilon_{c}<\varepsilon_{c,\lim}^{pl}\\ \Omega_{c}=\Omega_{cr}+\left(\Omega_{cu}-\Omega_{cr}\right)\;\exp\left(2\frac{\Omega_{cu}-1}{\varepsilon_{c,\lim}^{pl}-\varepsilon_{c,1}^{pl}}\;\frac{\varepsilon_{c}-\varepsilon_{c,\lim}^{pl}}{\Omega_{cu}-\Omega_{cr}}\right),\;\varepsilon_{c}>\varepsilon_{c,\lim}^{pl} \end{array}\right.$$

Here $\varepsilon_{c,\lim}^{pl}$ is the plastic strain at the transition point from power-law to exponential softening; $\Omega_{cu}$ is the residual relative stress at the transition from power-law to exponential softening.

Plastic deformation in standard concrete starts around 33–40% of its maximum strength. But experiments demonstrate that FC and fiber-reinforced concrete exhibit linear behavior around 85% of their maximum load. Therefore, the stress corresponding to the transition from elastic to plastic deformation is calculated as $\Omega_{ci}$ = 0.85. The remaining parameters are determined in accordance with recommendations [31,51,52].

The compressive strength of FC was determined experimentally and was 11.8 MPa. Plastic deformation at peak compressive stress

(7) $$\varepsilon_{c1}^{pl}=\varepsilon_{c1}-\frac{f_{c}}{E}$$

Here $f_{c}$ is a compressive strength, $\varepsilon_{c1}$ is a total strain, and *E* is Young’s modulus.

The formula determines plastic strain when transitioning from power law to exponential softening:

(8) $$\varepsilon_{c,\lim}^{pl}=\varepsilon_{c,\lim}-\frac{\sigma_{c,\lim}}{E}$$

Here $\varepsilon_{c,\lim}$ are the total deformations at the transition from power law to exponential softening.

The residual compressive relative stress is 0.2, determined from the stress–strain curve of FC.

## 3. Results and Discussion

### 3.1. Model Verification Using Experimental Data on Foam Concrete Prism Compression

**Figure 6:**
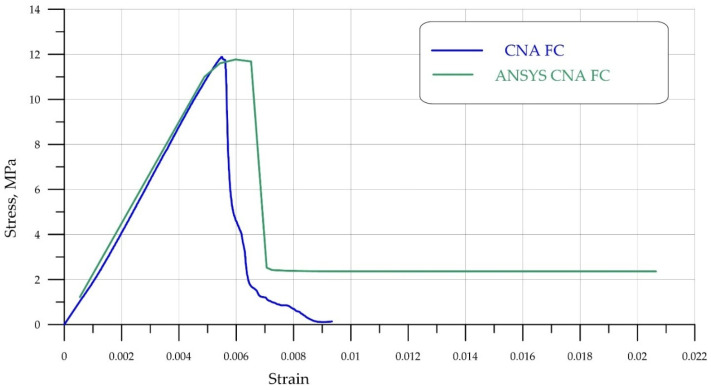
Comparison of stress–strain curves of FC obtained experimentally (blue curve) and based on numerical simulation (green curve).

**Figure 7:**
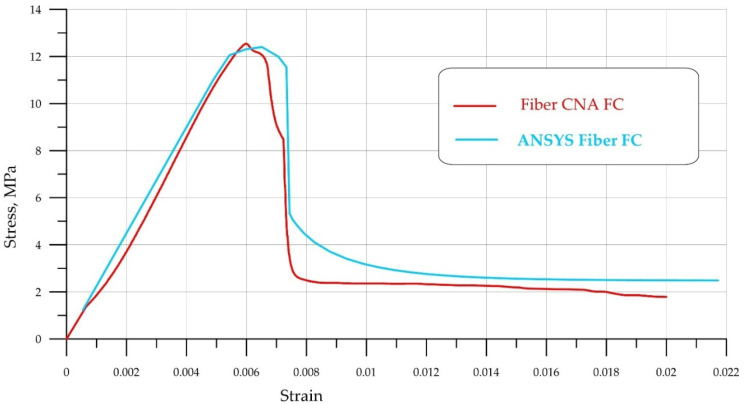
Comparison of stress–strain curves for FRFC obtained experimentally (red curve) and based on numerical simulation (blue curve).

A comparison of experimental data obtained from uniaxial compression tests of FC and FRFC specimens is shown in Figure 6 and Figure 7. Due to the symmetry of the problem, the numerical analysis was performed on a quarter-section of the prism under the following boundary conditions. A prism with dimensions *L* (height), *a* = *b* (width and length, respectively) (the dimensions of the prism base) occupies a position in XYZ space such that its height coincides with the X-axis, i.e., 0 ≤ *x* ≤ *L*. A displacement *δ* is applied to the upper face of the prism, *x = L*. The lower face, *x* = 0, is fixed along the X-axis in the X-direction, i.e., at *x* = 0, the displacement UX = 0. The prism face, *z* = *b*, is fixed in the Z-direction, i.e., at *z* = *b*, the displacement UZ = 0. The edge of the prism *y* = *b* is fixed in the Y direction, that is, when *y* = *b* the displacement is UY = 0.

**Figure 8:**
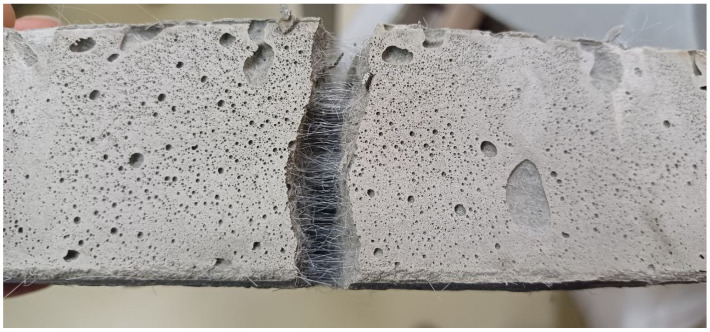
Failure mode of FRFC under tension.

Figure 6 and Figure 7 show that the stress–strain curves obtained numerically are in good agreement with the experimental data. In both cases, a brittle failure mode is observed, with the curve dropping sharply to a minimum value. It is also evident that FC without fibers (Figure 6, blue curve) quickly loses its load-bearing capacity, while FRFC (Figure 7, red curve) still resists failure due to the adhesion of the fibers to the matrix. Figure 8 shows a characteristic fracture pattern characteristic of FRFC failure.

**Figure 9:**
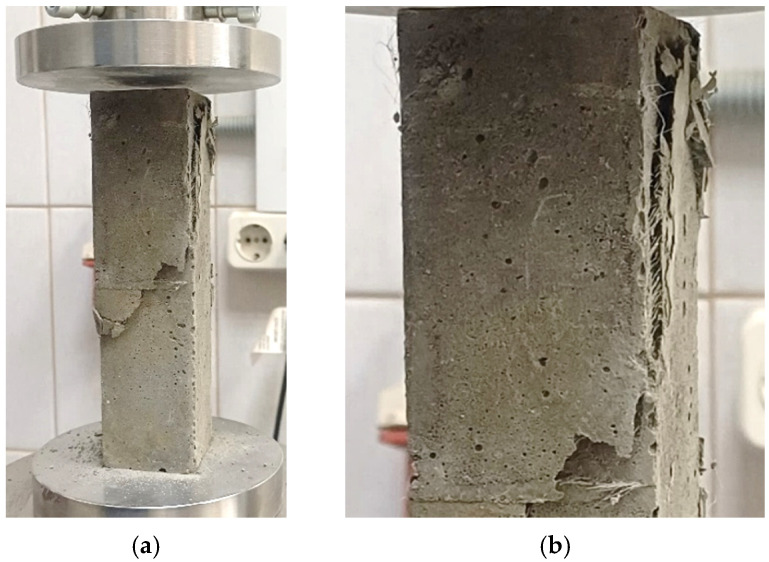
Compressive failure of FRFC: (**a**) specimen failure; (**b**) enlarged image showing fibers in the failure zone.

The resulting FEM model allowed for a detailed analysis of the stress–strain state of FRFC under compression. Figure 9 shows that FRFC failure under compression occurs when transverse strains exceed the ultimate value, due to the large difference between compressive and tensile strengths. FC reinforced with a complex nanomodifying additive behaves as a brittle material, exhibiting elastic behavior up to 85% of the ultimate load. This is followed by a stage of plastic deformation, which, due to the Poisson effect, ruptures the material in the transverse direction. The presence of polymer fibers restrains transverse deformation and enhances transverse strength. This is quantitatively reflected in Table 2. The compressive strength of the prisms was compared across six groups of specimens, with three specimens in each group. The results are presented in Table 3.

The average strength of FC was 11.8 MPa, while the average strength of FRFC was 14.07 MPa, representing a 20% increase in strength.

During FEM analysis, the FC matrix had a strength of 11.8 MPa, while the polypropylene fiber exhibited a bilinear curve: modulus of elasticity of 6000 MPa, yield strength of 320 MPa, and tangential hardening modulus of 500 MPa. The calculated compressive strength of fiber-reinforced concrete was 13.8 MPa, which is in good agreement with the experimental data.

### 3.2. Parametric Modeling of Foam Concrete Using the Menetrey-Willam Model

**Figure 10:**
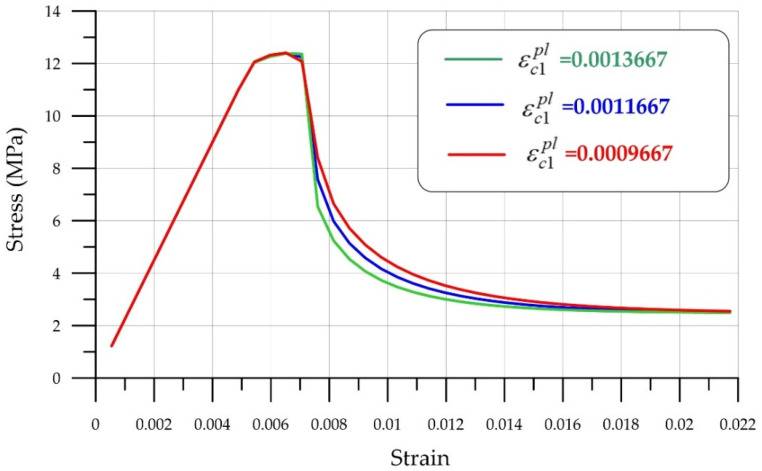
Effect of the plastic strength parameter $\varepsilon_{c1}^{pl}$ at uniaxial compressive strength.

Adjusting the parameters of the Menetrey-Willam model requires sufficiently precise laboratory equipment, as slight deviations lead to incorrect results. This section demonstrates the model’s sensitivity to parameter changes. Let us consider the influence of the parameter plastic strength at uniaxial compressive strength (Figure 10).

It is clear that this parameter $\varepsilon_{c1}^{pl}$ influences the shape of the descending curve and allows modeling the softening stage fairly closely to real brittle fracture conditions.

**Figure 11:**
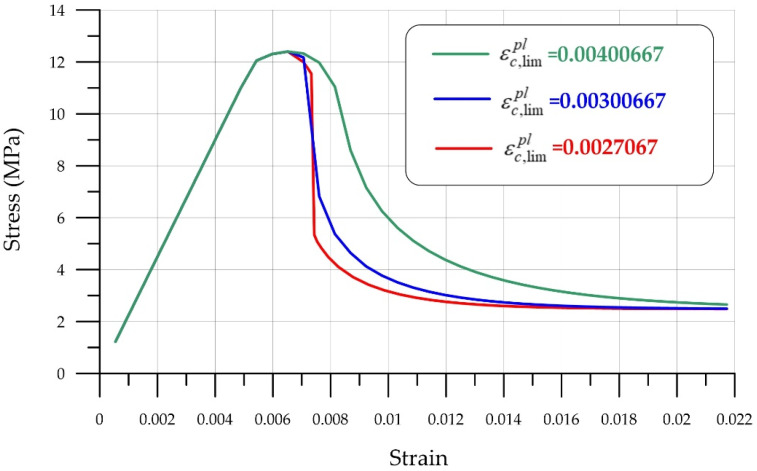
Influence of the deformation parameter $\varepsilon_{c,\lim}$ at transition from power law to exponential softening.

**Figure 12:**
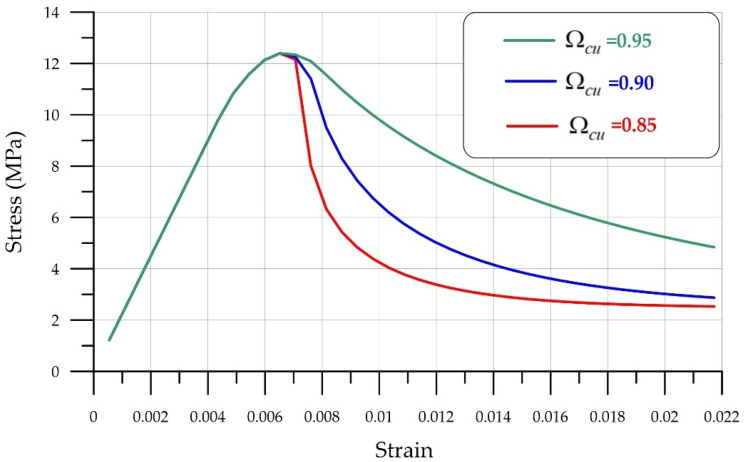
Effect of the residual relative stress parameter $\Omega_{cu}$ during the transition from power law to exponential softening.

Figure 11 and Figure 12 show graphs characterizing the effect of the deformation parameter $\varepsilon_{c,\lim}$ at the transition from power law to exponential softening and $\Omega_{cu}$ the residual relative stress parameter at the transition from power law to exponential softening.

**Figure 13:**
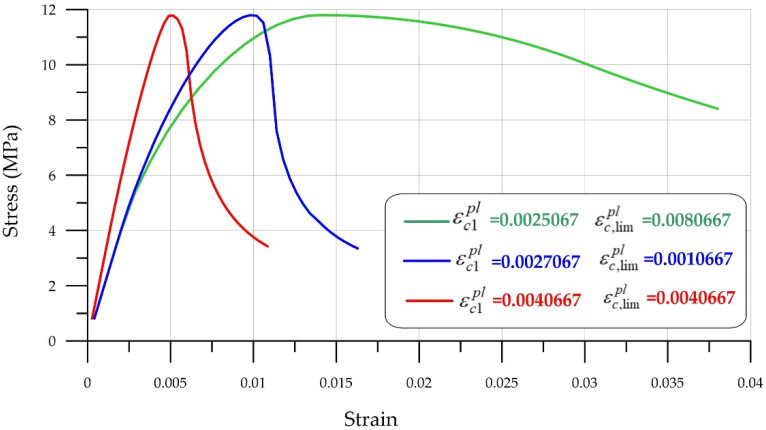
Effect of combination of parameters $\varepsilon_{c1}^{pl}$ and $\varepsilon_{c,\lim}$.

It is important to note that the parameters are interrelated, and changing one parameter must be accompanied by adjustments to others. Figure 13 shows the effect of parameter combinations $\varepsilon_{c1}^{pl}$ and $\varepsilon_{c,\lim}$ on the shape of the stress–strain curves.

### 3.3. Model Verification and Analysis of the Effect of Polymer Fiber on the Bending Stress–Strain

Since ANSYS generates a representative volume beam, the dimensions of which are significantly smaller than the experimental specimens, we will apply the principles of similarity theory to interpret the obtained data. The stress relationships must be identical, so we will consider the formula for bending stresses for a homogeneous and isotropic rectangular beam.

(9) $$\sigma =\frac{M_{x}}{W_{x}}\leq f_{c}$$

For a beam of length 2*L* and cross-sectional dimensions *a* = *b*, simply supported on two supports, where the distance between the supports is equal to *L*,

(10) $$\delta =\frac{F\;L^{3}}{48E\;I},\;I=\frac{ba^{3}}{12},\;a=b,\;L=4a,\Rightarrow \delta =\frac{12F\;64a^{3}}{48E\;a\;a^{3}}=\frac{16\;F}{E\;a}\Rightarrow \frac{F}{\delta }=\frac{Ea}{16}\;$$

In the experiment, *a* = 40 mm, while in the FEM model, it is 0.92054 mm. Thus, the scaling factor for deflection conversion is $\frac{a_{\exp}}{a_{FEM}}=43.452$.

**Figure 14:**
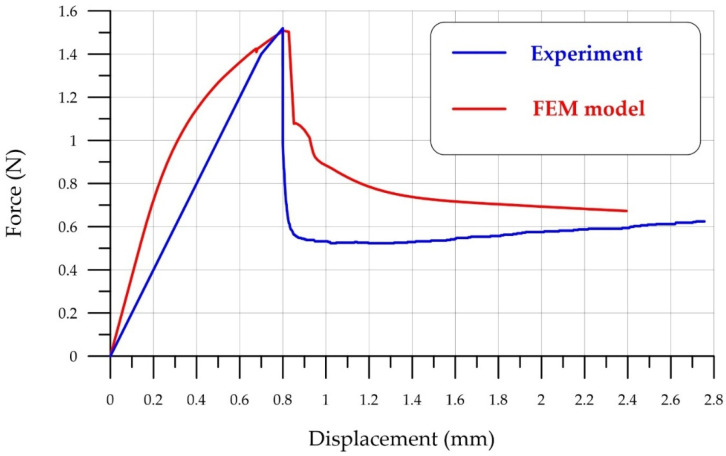
Comparison of experimental data and the FEM model for bending FRFC with polypropylene fibers.

Figure 14 shows a comparison of the experimental data with the FEM model. It is evident that the model qualitatively captures the brittle failure zone of FRFC well.

The close similarity of the descending stress–strain curve is noteworthy. Longitudinal deformations are restrained and complete specimen failure is resisted by polypropylene fibers, resulting in the flattening curve’s relatively long tail. The increase in flexural strength of FRFC is illustrated in Table 4.

### 3.4. Analysis of the Stress–Strain State of Fiber-Reinforced Foam Concrete in Bending

**Figure 15:**
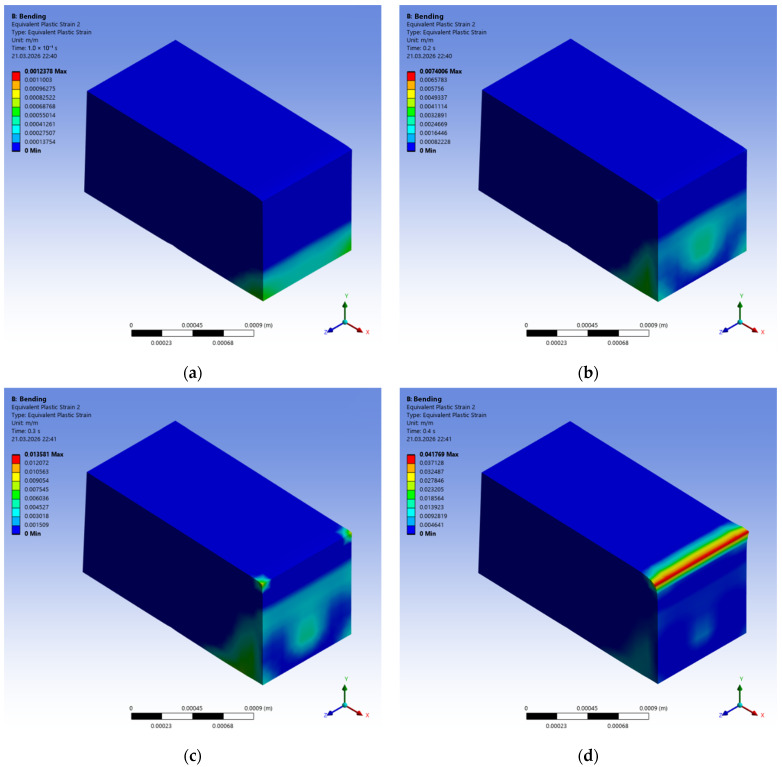
Sequential development of equivalent plastic strains (cracks) in FRFC with polypropylene fibers (% of maximum deformation): (**a**) at 10%; (**b**) 20%; (**c**) 30%; (**d**) 40%.

The progressive development of plastic deformations (cracks) in fiber-reinforced concrete with polypropylene fibers is shown in Figure 15.

It is evident that the crack initiates in the lower tensile chord (Figure 15a). The influence of the polypropylene fibers then creates a non-uniform stress–strain state along the height, in which part of the FC matrix fails, while part is restrained by the fibers (Figure 15b,c). Finally, in the failed specimen, the zone of greatest strain shifts toward the upper chord, while the lower chord is restrained only by the fibers.

**Figure 16:**
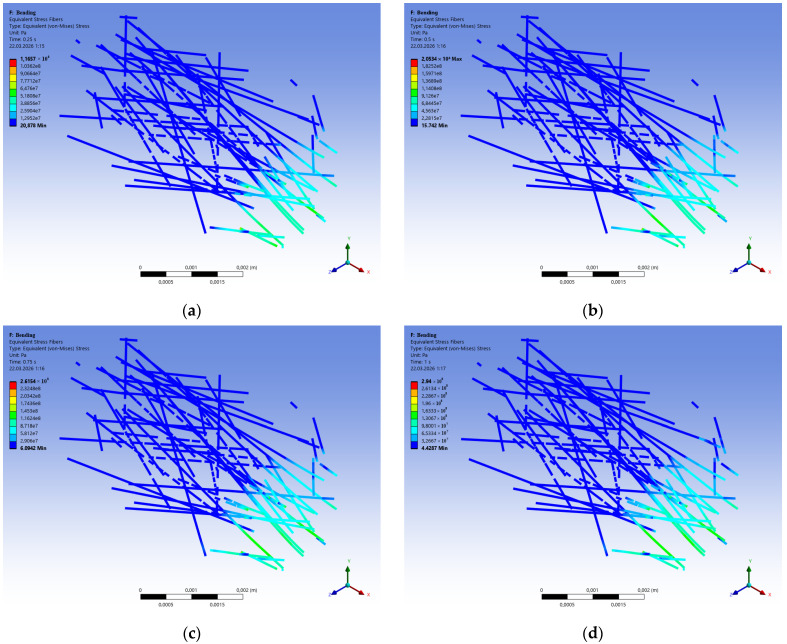
Sequential change in equivalent stresses in polypropylene fibers during bending of FRFC (% of maximum deformation): (**a**) at 25%; (**b**) 50%; (**c**) 75%; (**d**) 100%.

Figure 16 shows the sequential change in equivalent stresses in the polypropylene fibers during the bending of FRFC. Each image corresponds to the same loading stage, similar to Figure 15. It is recommended to analyze them together. Figure 15a shows the plastic strains in the matrix, and Figure 16a shows the equivalent stresses in the polypropylene fibers. It is clear that a crack is already beginning in the matrix, and the polypropylene is operating in the elastic deformation zone. Figure 15c,d and Figure 16c,d show active fracture of the lower matrix band, while the polypropylene fibers are experiencing stresses at the yield point.

Thus, Figure 16 shows that during the bending of FRFC, the fibers have a small reserve of strength, providing stable material strength at a concentration of 3.9 kg/m^3^.

### 3.5. Comparison of Different Fiber Types

**Figure 17:**
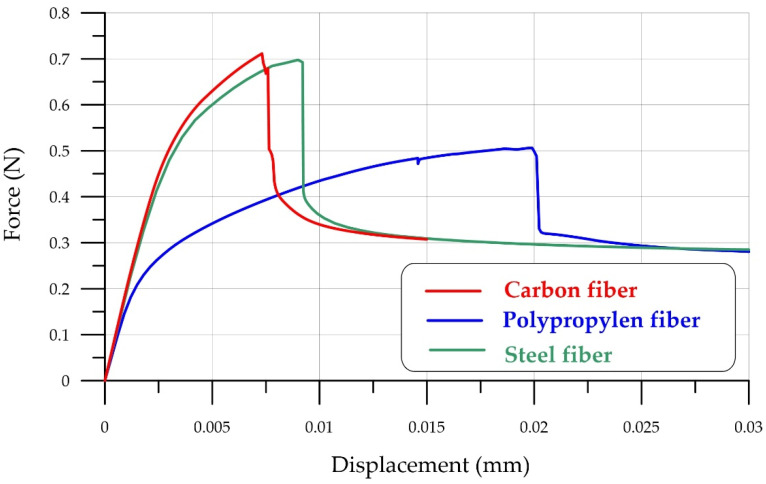
Comparison of force–displacement curves for FRFC with different fiber types: carbon, polypropylene, and steel.

Figure 15 and Figure 16 demonstrate that polypropylene fiber has a reserve of strength, so it is logical to consider how other polymer or steel fibers would behave. For a clear analysis, other fibers were considered at the same concentration and with the same geometric parameters. The only difference is in the mechanical properties of the fibers. Figure 17 compares the results of numerical modeling of FRFC bending with different fiber types: polypropylene, carbon, and steel.

**Figure 18:**
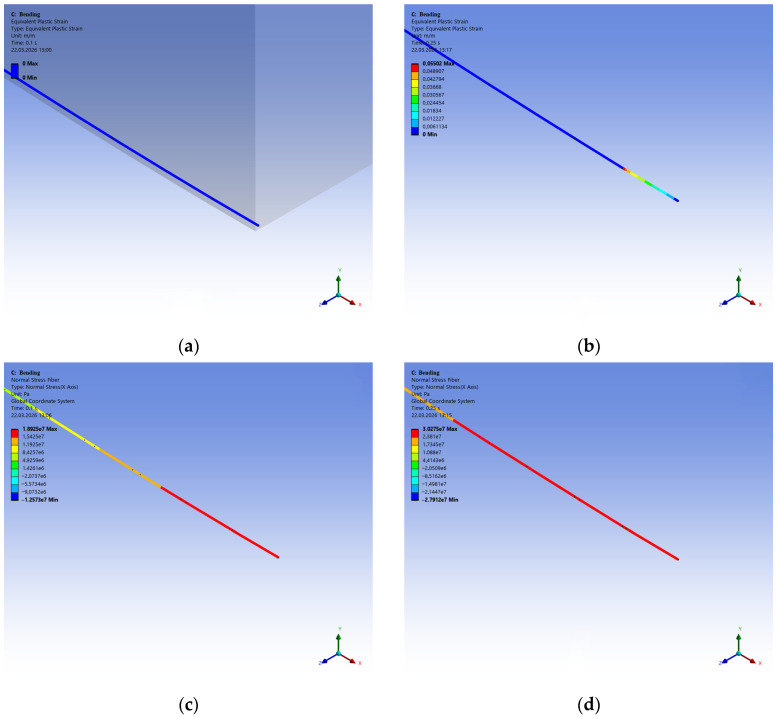
Strains and stresses in an individual polypropylene fiber under the highest load: (**a**,**c**) equivalent plastic strains and normal stresses at the 10% loading stage (no plastic deformation); (**b**,**d**) equivalent strains and normal stresses at the failure stage of the foam concrete.

Figure 17 compares force–displacement curves for three fiber types: carbon (*E* = 395 GPa), steel, and polypropylene. It is clear that fibers with a high modulus of elasticity and high strength provide greater fiber-reinforced concrete strength but reduce deformation properties. Given the difference in the modulus of elasticity and strength of the fibers and the concrete matrix, it can be concluded that high-modulus fibers are wasting their potential. When the matrix is destroyed, the fibers experience elastic stresses without reaching the yield strength. This is illustrated by Figure 18, which shows the equivalent plastic strains and equivalent von Mises stresses in an individual polypropylene fiber as an example.

Figure 18a,b shows that at the loading stage, when the matrix begins to destroy, the fiber does not undergo plastic deformation and operates in the elastic zone. After the lower chord of the FC matrix begins to fail, the fiber is in the plastic deformation stage and experiences stresses at the yield point, thereby restraining the overall failure of the FRFC.

Table 5 shows the properties of steel, carbon, and polypropylene fibers and their corresponding resilience and toughness moduli, calculated in accordance with ASTM 1609/1609M-10 [56]. For clarity of analysis, the fiber arrangement, concentration, and geometry have been kept the same as for polypropylene.

### 3.6. Evaluation of Fiber Adhesion Properties in Bending

The stress–strain state of fiber-reinforced concrete is characterized by non-uniform behavior due to the different properties of the fibers and FC. Differences in elastic moduli create different stresses in the fibers and concrete, while ensuring equal strains along their boundaries. Equal strains ensure good adhesion properties. Unequal strains result in fiber slippage relative to the concrete matrix.

**Figure 19:**
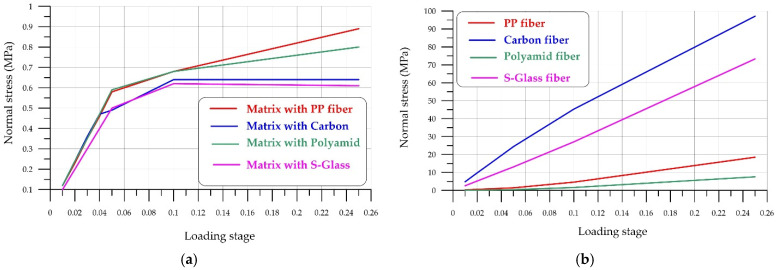
Stresses in the concrete matrix and in the loaded fiber itself at various loading stages: (**a**) stresses in the matrix; (**b**) stresses in the fiber.

Figure 19 shows the stresses in the FC matrix and in the loaded fiber itself at various loading stages. For consistency, loading stages are expressed as fractions of one, i.e., the maximum load.

The difference in normal stresses creates conditions for the loss of adhesion between the fiber and the matrix. In this case, the adhesion at the fiber–foam concrete interface is unable to effectively transmit the force, preventing the material’s strengthening properties from manifesting themselves. This difference in normal stresses is shown in Figure 17.

**Figure 20:**
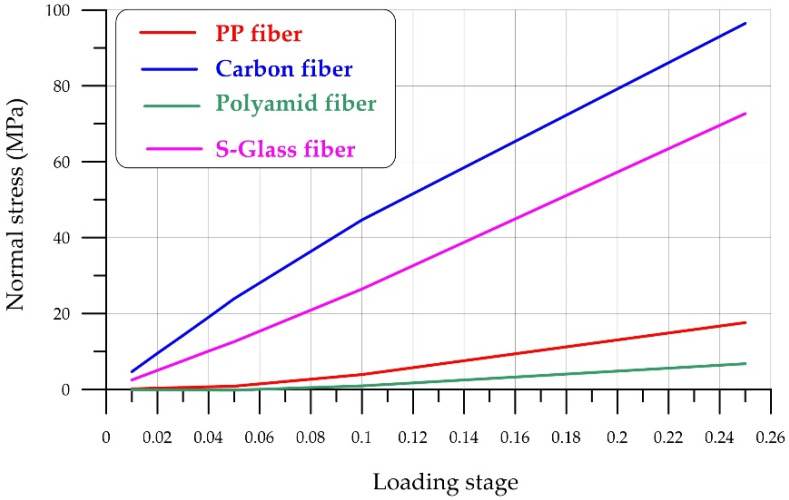
Stress difference at the interface between the matrix and fibers.

Figure 20 shows the stress difference in the fiber–foam concrete matrix bond zone. It is evident that low-modulus polymer fibers (polypropylene, polyamide) maintain bond strength with the matrix for a relatively long time, while high-modulus materials, due to their high elastic modulus, quickly create a stress difference and lose adhesion. Loss of adhesion occurs when the interface stress difference exceeds the maximum permissible adhesion stress. The magnitude of these stresses is determined experimentally, and this issue is addressed in [57,58,59,60]. Ref. [57] examines steel, polypropylene, and fiberglass fibers under different loading rates. The authors showed that steel fibers without an anchor hook provide bond strengths of 5 to 17 MPa, while polypropylene and polyethylene fibers provide bond strengths of up to 7 MPa. Carbon fibers [58,61] provide bond strength in the range of 5–7 MPa, basalt fiber up to 3.5 MPa. The data presented qualitatively characterize the adhesion strength indicators, since they were obtained under different temperature conditions and with different properties of the concrete matrix. At the same time, analyzing the ultimate values of fiber–concrete adhesion strength and taking into account the nature of the stress difference (Figure 20), one can conclude that the adhesion strength reserve of high-modulus fibers (steel, carbon) and widely used polypropylene and polyamide fibers, in which the adhesion force is realized until the destruction of the foam concrete, thereby ensuring good deformation properties of FRFC, is quickly exhausted.

A comparison with the results obtained in [62] shows that, for reinforced foam concrete, the compressive strength is in good agreement with the findings of [62]. Steel fibers contributed more to the compressive strength than glass fibers. The bond strength ratio between glass fibers and steel rods in FC specimens ranged from 37.8 to 89.3, which correlates with the present study, in which steel fibers improved strength by 31%. It should be noted that ref. [62] used hooked-end steel fibers. This increases adhesion and improves strength.

Work [63] also notes that dispersed reinforcement with both types of fibers improves the strength properties and durability of concrete under service conditions. However, the cost per cubic meter of concrete mix using polypropylene fibers is $12.5–$22.5 less than that of steel. Other advantages of polypropylene fibers are evident under complex loading conditions, such as bending, tension, and torsional bending. In these cases, the plasticity of polypropylene fibers leads to higher deformation properties of foam concrete.

## 4. Conclusions

This study proposes an approach to increasing the strength of foam concrete using a complex nanomodifying additive and the inclusion of polypropylene fiber. A FEM model for testing FRFC under compression and bending was constructed. Numerical and experimental studies showed the following:

(1) A new FRFC composition was developed containing a complex nanomodifying additive consisting of mineral components, nanoparticles, and a plasticizing additive in the following ratio: FA—80%; MS—18%; NA—2%. Polypropylene fiber was added to the composition at a content of 3.9 kg/m^3^.
(2) An FEM model based on the Menetrey-Willam approach was developed, and an analysis of the stress–strain state of FRFC under compression and bending was conducted. An algorithm for calibrating the parameters of the Menetrey-Willam model was proposed, and a comparison of the numerical and experimental data was conducted. The comparison revealed good qualitative and quantitative agreement between the results for active loading and softening of FRFC during failure.
(3) The stress–strain state of FRFC was analyzed with the inclusion of various types of fibers: steel, polypropylene, polyamide, carbon, and glass fiber. A comparison of the plastic deformations of the foam concrete matrix and fibers showed that polypropylene fiber provides good deformation properties of the composite. Steel or carbon fibers increase the strength of FRFC, but they degrade deformation properties and do not fully utilize the fiber’s potential.
(4) The adhesion properties of the fibers and the foam concrete matrix were analyzed. It was shown that high-modulus polymer fibers quickly lose their adhesive properties and degrade the deformation properties of the composite compared to polypropylene fibers.
(5) It has been experimentally established and numerically confirmed that CNA FRFC with polypropylene fiber increases compressive strength by 20% and flexural strength by 80%.

This study examines the strength properties of fiber-reinforced foam concrete and evaluates the contribution of different fiber types. However, several limitations should be noted. The study does not consider long-term strength or creep. The applied FEM analysis methods, based on the Menetrey-Willam model, lead to errors of approximately 15% when modeling brittle fiber-reinforced foam concrete. Methodologically, a quantitative assessment of the influence of a specific fiber type on the strength of fiber-reinforced foam concrete is based on a multi-step approach that requires time-consuming experimental and numerical studies, which also impacts the uncertainty of strength prediction.

## Figures and Tables

**Table 1:** Raw material properties.

Indicator	Actual Value
**Portland cement CEM I 42.5 N (PC) (CEMROS, Stary Oskol, Russia)**	
Specific surface area (m^2^/kg)	350
Normal density (%)	29
Setting time (hours-min)	3–10
Setting time (hours-min)	4–40
Compressive strength after 28 days (MPa)	52.3
Bending strength after 28 days (Mpa)	8.1
**Quartz sand (QS) (Subsoil, Samarskoye village, Russia)**	
Bulk density (kg/m^3^)	1348
Apparent density (kg/m^3^)	2575
The content of dust and clay particles (%)	0.12
**Fly ash (FA) (Novocherkassk State District Power Plant, Novocherkassk, Russia)**	
Bulk density (kg/m^3^)	932
**Microsilica (MS) (NLMK, Lipetsk, Russia)**	
Bulk density (kg/m^3^)	152
**Nanosized aluminum oxide (NA) particles (Shandong Tiancheng Chemical Co., Ltd., Yanzhou, China)**	
Average particle diameter (D50) (nm) NA	20–30
Specific surface area of particles (m^2^/g)	130
**Rospena (R) foaming agent (Rospena, Moscow, Russia)**	
Density (g/cm^3^)	1.1
Stability (hours)	1–3.5
**Polypropylene fiber (PF) (CEMMIX, Moscow, Russia)**	
Length (mm)	10–12
Diameter (µm)	34
Density (g/cm^3^)	0.91
Tensile Strength (MPa)	320
Elastic Modulus (GPa)	6
**Powdered plasticizing additive C-3 (CEMMIX, Moscow, Russia)**	
Appearance	Brown powder
pH	8 ± 1

**Table 2:** Experimental FC and FRFC compositions (per 1 m^3^).

Mixture Type	PC (kg/m^3^)	QS (kg/m^3^)	CNA (kg/m^3^)	PF (kg/m^3^)	Water (L)	Rospena (kg/m^3^)
Foam concrete	325.0	530	0	0	160	9.75
Fiber foam concrete	292.5	530	32.5	3.9	160	9.75

**Table 3:** Comparison of compressive strength of FC and FRFC samples.

Concrete Type	Sample Marking	Flexural Strength, MPa	
		Sample	Group
Foam concrete	FC1	11.10	11.8
	FC2	13.80	
	FC3	11.70	
	FC4	12.40	
	FC5	10.90	
	FC6	10.90	
Fiber foam concrete	FFC1	15.80	14.07
	FFC2	12.90	
	FFC3	13.60	
	FFC4	13.80	
	FFC5	14.30	
	FFC6	14.00	

**Table 4:** Results of experimental flexural tests of FC and FRFC.

Concrete Type	Sample Marking	Flexural Strength, MPa	
		Sample	Group
Foam concrete	FC1	1.84	1.8
	FC2	1.80	
	FC3	1.76	
	FC4	1.85	
	FC5	1.79	
	FC6	1.82	
Fiber foam concrete	FFC1	3.54	3.38
	FFC2	3.09	
	FFC3	3.52	
	FFC4	3.58	
	FFC5	3.25	
	FFC6	3.28	

The flexural strength of FRFC is 86.6% higher than FC.

**Table 5:** Specific properties of FC with and without fibers.

Fiber Type	Yield Strength, MPa	Ultimate Strength, MPa	Foam Concrete	
			Resilience Modulus, J × m^−3^	Toughness Modulus, J × m^−3^
FC without fibers	8	11.8	24.0	608
Polypropylene fiber	320	500	41.6	1877
Steel fibers	250	460	112.1	1402
Carbon 395	2000	2200	144.2	1798

## Data Availability

The original contributions presented in the study are included in the article, further inquiries can be directed to the corresponding author.

## Abbreviations

The following abbreviations are used in this manuscript:

PPF	Polypropylene fiber
CNA	Complex nanomodifying additive
FC	Foam concrete
FRFC	Fiber-reinforced foam concrete
CF	Carbon fiber
SF	Steel fiber

## References

[1] Pan G., Gong H., Sun Z., Chen L., Liu Z., Gao L. (2026). Towards high-performance low-carbon foam concrete: A review on multi-component synergy and pore structure optimization. J. Build. Eng..

[2] Song Q., Hu Y., Niu D., Zhao H., Xia J., Bao J., Li R., Wang Y., Xue S., Tang X. (2026). Research on durability of foam concrete: A systematic review. J. Build. Eng..

[3] Liu J., Liu Y., Ma X., Xu H., Wu X., Liu M., Zhuge Y. (2025). Thermal performance optimisation of foam concrete for energy-efficient construction: A state-of-the-art review. J. Build. Eng..

[4] Stel’makh S.A., Shcherban’ E.M., Beskopylny A.N., Mailyan L.R., Meskhi B., Shilov A.A., Mailyan A.L., Zakieva N.I., Chernil’nik A., El’shaeva D. (2023). Structural Formation and Properties of Eco-Friendly Foam Concrete Modified with Coal Dust. J. Compos. Sci..

[5] Iozzino F., Fragnito A., Mauro G.M., Roselli C. (2026). Preliminary Optimization of Steady-State and Dynamic Thermal Performance of 3D Printed Foamed Concrete. Thermo.

[6] Beskopylny A.N., Stel’makh S.A., Shcherban’ E.M., Shakhalieva D.M., Chernil’nik A., Shcherban’ N., Vyalikov I., Budovskiy A. (2026). Mechanical Properties and Microstructure of Environmentally Friendly Foam Concrete with Fly Ash Modified with Micro- and Nano-SiO_2_. Materials.

[7] Rajpurohit K., Shaikh S.A., Pandey A.K., Bagla H.K. (2025). Synthetic polymers and nanostructured materials additives for engineered cementitious materials: Plausible route for recycled polymer utilization. Hybrid Adv..

[8] Beskopylny A.N., Stel’makh S.A., Shcherban’ E.M., Saidumov M.S., Abumuslimov A., Mezhidov D., Wang Z. (2025). Eco-Friendly Foam Concrete with Improved Physical and Mechanical Properties, Modified with Fly Ash and Reinforced with Coconut Fibers. Constr. Mater. Prod..

[9] Behera D., Liu K.-Y., Rachman F., Worku A.M. (2025). Innovations and Applications in Lightweight Concrete: Review of Current Practices and Future Directions. Buildings.

[10] Ahmed S.A., Ebrahem E., El-Feky M.S. (2024). Achieving sustainable performance: Synergistic effects of nano-silica and recycled expanded polystyrene in lightweight structural concrete. Sci. Rep..

[11] Mohtadi A., Ghomeishi M., Dehghanbanadaki A. (2025). Machine learning-based prediction of thermal conductivity in foamed concrete: Influence of nano-microsilica compounds and air content using XGBoost. Ain Shams Eng. J..

[12] Mahajan D.S., Muhammad S. (2026). Effect of alkali activation and foaming on the chemistry, microstructure, and mechanical properties of copper slag–based sustainable cellular concrete blocks. Mater. Chem. Phys..

[13] Wu C., Jierula A., Tang F., Wu J., Oh T. (2026). Preparation of foamed concrete with bentonite instead of cement: Compressive strength and durability. Constr. Build. Mater..

[14] Zhou G., Su R.K.L. (2023). A Review on Durability of Foam Concrete. Buildings.

[15] Li R., Yin Z., Lin H. (2023). Research Status and Prospects for the Utilization of Lead–Zinc Tailings as Building Materials. Buildings.

[16] Wei C., Liu X., Zhan Z., Wu P. (2024). Utilization of solid wastes for aerated concrete preparation: Mechanical properties and microstructural analysis. J. Build. Eng..

[17] Meskhi B., Beskopylny A.N., Stel’makh S.A., Shcherban’ E.M., Mailyan L.R., Beskopylny N., Chernil’nik A., El’shaeva D. (2022). Insulation Foam Concrete Nanomodified with Microsilica and Reinforced with Polypropylene Fiber for the Improvement of Characteristics. Polymers.

[18] Li M., Hu Y., Rao L., Jiang L., Li J., Zhou S., Sun H., Peng S., Pang X., Chen Y. (2025). Numerical Analysis on Mechanical Properties of Different Fiber-Reinforced Cold-Formed Steel–Concrete Composite Corner Columns. Polymers.

[19] Al Tekreeti M., Bahadori-Jahromi A., Room S., Tariq Z. (2026). Optimized Machine Learning Models for Predicting Compressive, Tensile, and Flexural Strengths of Multi-Fiber Recycled Aggregate Concrete. J. Compos. Sci..

[20] Ünverdi M., Bayqra S.H., Kaya Y., Özen S., Mardani A., Ramyar K. (2026). Mechanical Performance, Statistical Optimization, and Environmental Impact of Roller-Compacted Concrete Reinforced with Waste and Industrial Fibers. Buildings.

[21] Jadooe A., Whwah M.S., Al-Hussainy H.A., Kaishesh A.J., Pinto H.A.S., Bernardo L.F.A., Dulaimi A. (2026). Effects of Silica Fume, Perlite, and Polypropylene Fibers on the Mechanical Properties of Lightweight Polystyrene Concrete Composite. J. Compos. Sci..

[22] Caggiano A., Pepe M., Xargay H., Martinelli E. (2020). Analytical Modeling of the Postcracking Response Observed in Hybrid Steel/Polypropylene Fiber-Reinforced Concrete. Polymers.

[23] Li F., Jin S., Cheng P., Wang Z., Yang Z. (2024). Study on Mechanical Properties and Carbon Emission Analysis of Polypropylene Fiber-Reinforced Brick Aggregate Concrete. Polymers.

[24] Zhao Q., Yang Z., Zhang X., Xia Z., Xiong K., Yan J. (2025). An Experimental Study on the Mechanical Properties and ANN-Based Prediction of a Tensile Constitutive Model of ECCs. Polymers.

[25] Ji Y., Pei Z. (2023). Investigation of Mechanical Properties of Ultra-High-Performance Polyethylene-Fiber-Reinforced Recycled-Brick-Aggregate Concrete. Polymers.

[26] Jueyendah S., Ağcakoca E. (2026). An Interpretable Ensemble Machine Learning Framework for Predicting the Ultimate Flexural Capacity of BFRP-Reinforced Concrete Beams. Polymers.

[27] Sun H., Shen J., Su X., Sun Y., Jiang H., Cui Z., Yang X. (2026). Fatigue characteristics and mechanical evaluation of basalt fiber reinforced composite material concrete under freeze-thaw cycles. Sci. Rep..

[28] Xu X., Li Y., Liang R. (2026). Mechanical Behavior and Modeling of Polypropylene Fiber-Reinforced Cemented Tailings Interface with Granite Under Shear Loading: Effects of Roughness and Curing Time. Buildings.

[29] Guo P., Zhang H., Ge E., Lin M., Jia H., Zhang Y., Fan X. (2026). Optimization and Performance Study of 3D Printed Concrete Mixture for Underground Utility Tunnels. Buildings.

[30] Li S., Qiu S., Zheng H., Yu H. (2026). Influence of fibers on the multiscale properties of fly ash and slag based geopolymer foam concrete. Constr. Build. Mater..

[31] Beskopylny A.N., Stel’makh S.A., Shcherban’ E.M., Shakhalieva D.M., Chernil’nik A., Panfilov I., Beskopylny N., Özkılıç Y.O. (2026). Finite Element Modeling and Experimental Study of Foam Concrete and Polystyrene Concrete. Buildings.

[32] Harshavardhan G., Thiagu H., Ravichandran P.T. (2026). The Experimental and Numerical Behaviour of Short Columns with Foam Concrete Using Steel Fibre and Polypropylene Fiber. Proceedings of the International Conference on Civil Engineering Innovative Development in Engineering Advances (ICC IDEA—2025), Lecture Notes in Civil Engineering.

[33] Sriram S., Thiagu H. (2026). Analytical and Experimental Study of Wall Panel with Steel and Polypropylene Fibre in Foam Concrete. Proceedings of the International Conference on Civil Engineering Innovative Development in Engineering Advances (ICC IDEA—2025), Lecture Notes in Civil Engineering.

[34] Khan M., Shakeel M., Khan K., Akbar S., Khan A. (2022). A Review on Fiber-Reinforced Foam Concrete. Eng. Proc..

[35] Thangavel R., Shukla S.K., Madhavan M.K. (2026). Fiber-Reinforced Foam Concrete Using Quarry Micro Fines and Sugarcane Bagasse Ash: A Box–Behnken Design Optimization and Performance Assessment. Sustainability.

[36] Amran M., Fediuk R., Vatin N., Huei Lee Y., Murali G., Ozbakkaloglu T., Klyuev S., Alabduljabber H. (2020). Fibre-Reinforced Foamed Concretes: A Review. Materials.

[37] Beskopylny A.N., Shcherban’ E.M., Stel’makh S.A., Mailyan L.R., Meskhi B., Varavka V., Chernil’nik A., Pogrebnyak A. (2023). Improved Fly Ash Based Structural Foam Concrete with Polypropylene Fiber. J. Compos. Sci..

[38] Klyuev S.V., Khezhev T.A., Pukharenko Y.V., Klyuev A.V. (2018). Fiber Concrete on the Basis of Composite Binder and Technogenic Raw Materials. Mater. Sci. Forum..

[39] Fediuk R.S., Lesovik V.S., Svintsov A.P., Mochalov A.V., Kulichkov S.V., Stoyushko N.Y., Gladkova N.A., Timokhin R.A. (2018). Self-compacting concrete using pretreatmented rice husk ash. Mag. Civ. Eng..

[40] Lesovik V., Voronov V., Glagolev E., Fediuk R., Alaskhanov A., Amran Y.H.M., Murali G., Baranov A. (2020). Improving the behaviors of foam concrete through the use of composite binder. J. Build. Eng..

[41] Ecemis A.S., Karalar M., Beskopylny A.N., Stel’makh S.A., Shcherban’ E.M., Aksoylu C., Madenci E., Özkılıç Y.O. (2025). The Influence of Fiber-Form Waste Tire Aggregates on the Flexural Strength, Ductility, and Energy Dissipation of Pultruded GFRP–Rubberized Concrete Hybrid Beams. Polymers.

[42] Beskopylny A.N., Shcherban’ E.M., Stel’makh S.A., Chernilnik A., Elshaeva D., Ananova O., Mailyan L.D., Muradyan V.A. (2025). Optimization of the Properties of Eco-Concrete Dispersedly Reinforced with Hemp and Flax Natural Fibers. J. Compos. Sci..

[43] Shcherban’ E.M., Stel’makh S.A., Beskopylny A.N., Meskhi B., Efremenko I., Shilov A.A., Vialikov I., Ananova O., Chernil’nik A., Elshaeva D. (2024). Composition, Structure and Properties of Geopolymer Concrete Dispersedly Reinforced with Sisal Fiber. Buildings.

[44] Klyuev S.V., Klyuev A.V., Ayubov N.A., Fediuk R.S., Levkina E.V. (2025). Finite Element Design and Analysis of Sustainable Mono-Reinforced and Hybrid-Reinforced Fiber geopolymers. Adv. Eng. Res..

[45] Kondratieva T.N., Chepurnenko A.S. (2024). Prediction of Rheological Parameters of Polymers by Machine Learning Methods. Adv. Eng. Res..

[46] Yang J., Guo Y., Wu Y., Zhang J. (2024). Dynamic Response of Fiber–Metal Laminates Sandwich Beams under Uniform Blast Loading. Materials.

[47] Luo Z., Yu N., Li Z., Wang Y., Cai Y., Liu G. (2025). Crash test and evaluation of EPS foam-filled UHPC sandwich retrofitted concrete barriers. Eng. Struct..

[48] Huang J.-Q., Dan M.-L., Chong X., Jiang Q., Feng Y.-L., Wang Y.-W. (2025). Out-of-plane shear performance of textile reinforced concrete sandwich panel: Numerical analysis and parametric study. Structures.

[49] Ashrafi E., Farzam M. (2025). An Experimental Approach to Lightweight Aggregate Concrete Material Modeling Parameters Under Cyclic and Biaxial Loadings. Int. J. Concr. Struct. Mater..

[50] Xu S., Fu P., Liu Y., Huang T., Wang X., Li Y. (2025). Experimental and 3D Simulation Research on the Mechanical Properties of Cold-Bonded Fly Ash Lightweight Aggregate Concrete Exposed to Different High Temperatures. Materials.

[51] Dmitriev A., Novozhilov Y., Mikhalyuk D., Lalin V. (2020). Calibration and Validation of the Menetrey-Willam Constitutive Model for Concrete. Constr. Unique Build. Struct..

[52] Santos L.M., Lima P.R.L., Santos G.J.B. (2025). Menetrey-Willam numerical model for analysis of fiber reinforced concrete beams. Rev. IBRACON Estrut. Mater..

[53] (1981). Interstate Standard. Cements. Methods for Determining Flexural and Compressive Strength.

[54] Testing Hardened Concrete—Part 3: Compressive Strength of Test Specimens. iTeh Standards: Etobicoke, ON, Canada, 2019.

[55] (2024). Concretes. Methods of Prismatic, Compressive Strength, Modulus of Elasticity and Poisson’s Ratio Determination.

[56] (2010). Standard Test Method for Flexural Performance of Fiber Reinforced Concrete (Using Beam with Third-Point Loading).

[57] Li L., Li X., Wang B., Tao J., Shi K. (2025). A review on interfacial bonding behavior between fiber and concrete. J. Build. Eng..

[58] Ji H., Lee D., Kim J.-H., Yang S.-B., Park B., Kwon D.J. (2026). Identification of critical factors governing the failure behavior of fiber-reinforced composites: Proposal of a weakness parameter. Compos. Part B Eng..

[59] Li V.C., Wu H., Chan Y. (1996). Effect of plasma treatment of polyethylene fibers on interface and cementitious composite properties. J. Am. Ceram. Soc..

[60] Francioso V., Moro C., Castillo A., Velay-Lizancos M. (2021). Effect of elevated temperature on flexural behavior and fibers-matrix bonding of recycled PP fiber-reinforced cementitious composite. Constr. Build. Mater..

[61] Huang H., Luo J., Peng C., Sun T., Deng T., Hu J., Kasimova G.A., Nurmirzayev A.D.U., Dongshuai H., Wei J. (2023). Interfacial bond between modified micro carbon fiber and high-strength cement paste in UHPC: Bond-slip tests and molecular dynamic simulation. Cem. Concr. Compos..

[62] Abd S.M., Hadi R., Abdal S., Shamim S., Najm H.M., Sabri M.M.S. (2023). Effect of Using Glass Fiber Reinforced Polymer (GFRP) and Deformed Steel Bars on the Bonding Behavior of Lightweight Foamed Concrete. Buildings.

[63] Kos Ž., Kroviakov S., Kryzhanovskyi V., Hedulian D. (2022). Strength, Frost Resistance, and Resistance to Acid Attacks on Fiber-Reinforced Concrete for Industrial Floors and Road Pavements with Steel and Polypropylene Fibers. Materials.

